# Access to timely formal dementia care in Europe: protocol of the Actifcare (ACcess to Timely Formal Care) study

**DOI:** 10.1186/s12913-016-1672-3

**Published:** 2016-08-23

**Authors:** Liselot Kerpershoek, Marjolein de Vugt, Claire Wolfs, Hannah Jelley, Martin Orrel, Bob Woods, Astrid Stephan, Anja Bieber, Gabriele Meyer, Knut Engedal, Geir Selbaek, Ron Handels, Anders Wimo, Louise Hopper, Kate Irving, Maria Marques, Manuel Gonçalves-Pereira, Elisa Portolani, Orazio Zanetti, Frans Verhey, the Actifcare Consortium, Frans Verhey, Marjolein de Vugt, Claire Wolfs, Ron Handels, Liselot Kerpershoek, Gabriele Meyer, Astrid Stephan, Anja Bieber, Anja Broda, Gabriele Bartoszek, Bob Woods, Martin Orrell, Hannah Jelley, Anders Wimo, Anders Sköldunger, Britt-Marie Sjölund, Knut Engedal, Geir Selbaek, Mona Michelet, Janne Rosvik, Siren Eriksen, Kate Irving, Louise Hopper, Rachael Joyce, Manuel Gonçalves-Pereira, Maria J. Marques, M. Conceição Balsinha, Ana Machado, Orazio Zanetti, Elisa Portolani

**Affiliations:** 1 Maastricht University, Maastricht, Netherlands; 2 Bangor University, Bangor, UK; 3 UCL, London, UK; 4 Martin-Luther University Halle-Wittenberg, Halle, Germany; 5 Oslo University Hospital, Oslo, Norway; 6 Karolinska Institutet, Solna, Sweden; 7 Dublin City University, Dublin, Ireland; 8 CEDOC, Nova Medical School | Faculdade de Ciências Médicas, Universidade Nova de Lisboa, Lisbon, Portugal; 9 Alzheimer’s Research Unit-Memory Clinic, IRCCS “Centro S.Giovanni di Dio, Brescia, Italy

**Keywords:** Dementia, Formal care, Service use, Needs

## Abstract

**Background:**

Previous findings indicate that people with dementia and their informal carers experience difficulties accessing and using formal care services due to a mismatch between needs and service use. This mismatch causes overall dissatisfaction and is a waste of the scarce financial care resources. This article presents the background and methods of the Actifcare (ACcess to Timely Formal Care) project. This is a European study aiming at best-practice development in finding timely access to formal care for community-dwelling people with dementia and their informal carers. There are five main objectives: 1) Explore predisposing and enabling factors associated with the use of formal care, 2) Explore the association between the use of formal care, needs and quality of life and 3) Compare these across European countries, 4) Understand the costs and consequences of formal care services utilization in people with unmet needs, 5) Determine the major costs and quality of life drivers and their relationship with formal care services across European countries.

**Methods:**

In a longitudinal cohort study conducted in eight European countries approximately 450 people with dementia and informal carers will be assessed three times in 1 year (baseline, 6 and 12 months). In this year we will closely monitor the process of finding access to formal care. Data on service use, quality of life and needs will be collected.

**Discussion:**

The results of Actifcare are expected to reveal best-practices in organizing formal care. Knowledge about enabling and predisposing factors regarding access to care services, as well as its costs and consequences, can advance the state of the art in health systems research into pathways to dementia care, in order to benefit people with dementia and their informal carers.

## Background

Approximately 60 % of persons with dementia live at home. They have an increased need of care as the disease progresses. In many countries people with dementia are encouraged to live at home as long as possible, as it is assumed that quality of life is better at home than in institutions and this could also decrease the financial burden of dementia [1]. Several national and international organizations such as Alzheimer’s Disease International (ADI) and Alzheimer Europe, have adopted strategies to promote timely recognition of dementia (European Parliament 2011, (http://www.alzheimer-europe.org/). A timely diagnosis is regarded as necessary to enable improvements in dementia care. It allows stakeholders to collaborate in making important decisions regarding post-diagnostic care. Timely access to dementia care services is considered crucial to reduce health care costs e.g., to increase the quality of life for patients, to reduce informal caregiver burden, and by better coordinating nursing home placement [2]. ‘Timely’ is preferred to ‘early’ in this context, emphasizing that it is personally tailored and aimed at reducing both the risk of overtreatment as well as undertreatment.

**Fig. 1:**
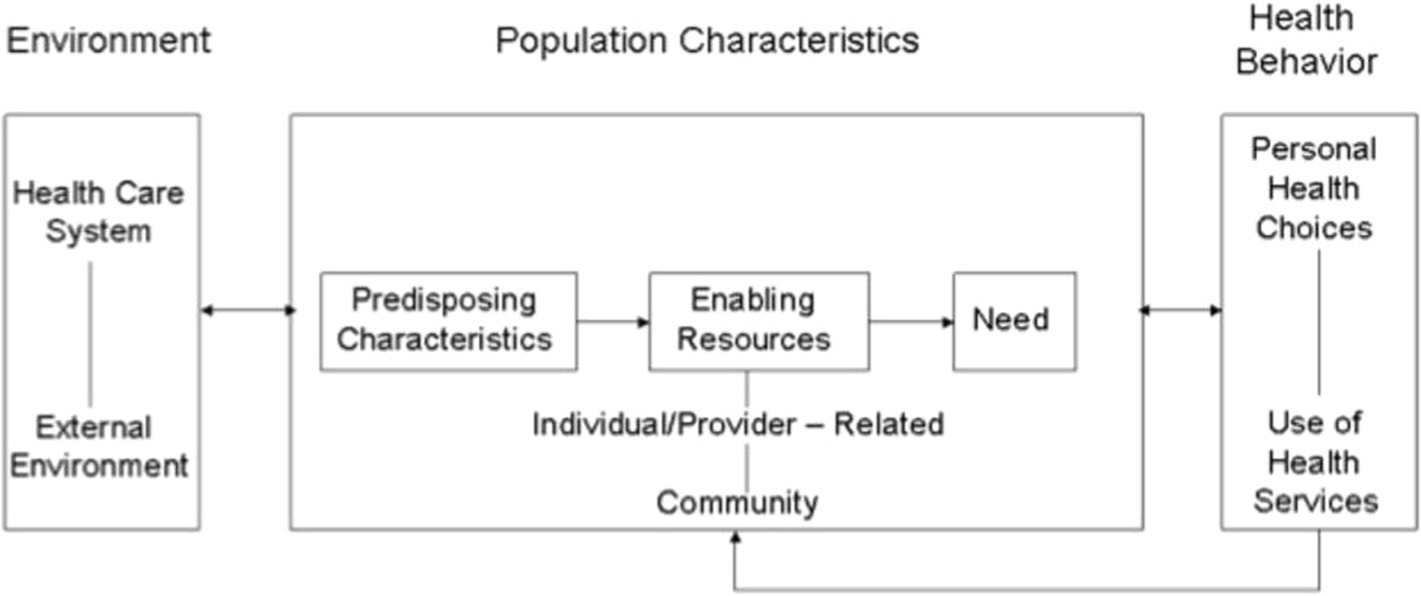
Andersen Behavioural Model of Health Services Use. Graphic representation of the model [6]

Previous findings indicate that people with dementia and their informal carers experience difficulties accessing and working with community care services, even when having a diagnosis of dementia [3, 4]. This can put increasing pressure on them, which might lead to admission to a residential home simply because the appropriate support is not in place [5]. If such a mismatch between needs and service use occurs there is overall dissatisfaction for the service user and a waste of the scarce financial care resources. In these times where the financial burden of dementia should be decreased by encouraging people with dementia to live at home as long as possible, other efforts to restrain budget are of interest too. Economic evaluation of formal care service use is thus a crucial task. Factors that influence the access to and use of formal care can be explored with the Behavioural Model of Health Service Use by Andersen and colleagues [6] (see Fig. 1). This model describes predisposing and enabling factors in relation to needs and service use. The main deduction is that before services are being used, various factors positively influence patients and their informal carers to use services (predisposing variables), while other factors enable service use (enabling factors), and other variables determine the need for care (need variables). Predisposing variables include demographics (age, gender, marital status), socio-structural variables such as education and ethnicity, and health beliefs for example about disease and care. Enabling factors are resources either supporting or impeding service use (waiting lists, health insurance coverage). Need variables consist of the impairments that require service, e.g. type of illness. The relation between these variables is complex, and could change during the progress of dementia, as needs are constantly changing [7].

*Figure**1**: Andersen Behavioural Model of Health Service Use* [8]*.*

The Andersen model can be used to understand access to and use of services by identifying associations between service use and a broad spectrum of predisposing and enabling variables, while controlling for need. The differences between countries regarding equity in which services are accessed and delivered have not yet been studied, despite the critical nature of this information for understanding the current health care systems. Well-organized access to formal care is especially important in the middle stage of dementia, as increased care is needed in this stage.

The Actifcare (Access to timely formal care) study focuses on this middle stage of dementia, which makes it innovative in contrast to previous dementia studies that have focused predominantly on early or later stages. Actifcare builds on a previous European Commission Framework Programme project called Right Time Place Care (RTCP) [9] and focuses on people who are not using formal care, but are most likely to start in the near future. This enables a specific evaluation of the process of accessing formal care.

This protocol focuses on the part of the Actifcare study that aims to increase our understanding of why people with dementia and their informal carers use, or fail to use formal care services across Europe, and how the use of formal care is experienced. In Actifcare formal care includes home nursing care, day care service, community or long-term medical care, nursing and social care structures. It excludes domestic home help, housekeepers, volunteers, support groups, transport services and meal programs. The present state of the art concerning access to timely formal care for people with dementia and their informal carers will be envisioned. We want to explore the reasons behind (non)-use of formal care by learning from experiences of people with dementia and their informal carers. Through different methods of data collection we aim to identify best-practice strategies regarding access to formal care for this vulnerable group. We will also use our large international database to validate some new measures during this study. Knowledge about enabling and predisposing factors regarding access to care services as well as its costs and consequences can advance the state of the art in health systems research into pathways to dementia care, in order to benefit people with dementia and their informal carers.

The main objectives are the following:

1. Exploring the predisposing and enabling factors that are associated with the use of formal care services;
2. Exploring the association between the use of formal care, needs and quality of life in people with dementia and their informal carers;
3. Comparing these across different European countries;
4. Understanding the costs and consequences of formal care services utilization in people with unmet needs in Europe;
5. Determining the major costs and quality of life drivers and the relation with formal care services across European countries;
6. Validation of the relatively new ICECAP-O instrument and the CarerQol instrument for the assessment of quality of life in relation to the timing of formal care in Europe.

## Methods/Design

### Overall methodology

To achieve these objectives a longitudinal cohort of patients will be recruited in eight countries. Themes, which are obtained in focus groups and expert interviews preceding this longitudinal study, will form the basis for in-depth semi-structured interviews.

### Design

A prospective cohort study design is adopted and is conducted in eight European countries (Germany, Netherlands, Sweden, Norway, Ireland, United Kingdom, Portugal and Italy). Baseline assessments started in November 2014 and follow-up measurements are planned after 6 and 12 months. Last patient out is expected in June 2016.

### Participants

The study aims to assess 480 dyads (60 per country) representing a cohort of community-dwelling people with dementia and their informal carers. Due to the exploratory nature of this study no power calculation is necessary. Eligibility criteria are described in Table 1. Participation is restricted to mentally competent people with dementia. Only people with dementia and their carers who provided informed consent participate. The carer and the person with dementia both sigh a separate informed consent form, after they had sufficient time to read the form and ask questions if needed. Participants are recruited from various settings, e.g. general practices, memory clinics, casemanagers and community mental health teams. In addition advertisements are placed in local and various national newspapers.

**Table 1:** Eligibility criteria for dyad selection

- The patient has a diagnosis of dementia meeting DSM IV TR criteria following an assessment by a clinical professional.	
- The person with dementia has a Clinical Dementia Rating indicating mild or moderate degree of dementia (i.e. scores 1 or 2) or scores 24 or less on the MMSE.	
- The patient is not receiving regular assistance from a paid worker with personal care, on account of his/her dementia, such as help with dressing/undressing; washing/ bathing/ showering; toileting; feeding/drinking; taking medication. (Note: ‘regular’ is defined as at least once per week; ‘paid worker’ includes those paid by health and social care services and those paid direct by the person and his/her family).	
- A professional judges that additional assistance with personal care is likely to be considered/required within 1 year.	
- The person with dementia has a carer who is able and willing to participate and is in contact at least once per week. The carer does not have to be residing with the carer, they could be a relative, friend or neighbour in regular contact.	
*Exclusion criteria*	
- The person with dementia or their carer is not able to complete the assessments due to communication/language/hearing/understanding/literacy problems that cannot be compensated for.	
- The person with dementia or their carer has a terminal condition or comorbidities (including long-standing severe mental illness) contributing to a significant level of disability	
- The person with dementia or their carer has a life-long learning disability or severe physical impairment that would prevent them from being able to complete the assessments.	
- The person with dementia resides in a care home or nursing home or has been resident in a care home or nursing home (e.g. for respite) during the previous 6 months.	
- The person with dementia has a diagnosis of alcohol-related dementia or of Huntington’s disease.	

### Measures

Table 2 summarizes the outcome measures, which were selected through careful consideration of psychometric properties and clinical utility. Questionnaires that were not available in all languages were translated and back translated via a translation protocol to ensure validity.

**Table 2:** Measurement instruments

Measurement instruments		
Variable	Measure	Assessed by
People with dementia		
Socio-demographics	Datasheet^a^	PwD
Cognition	MMSE	PwD
Service use	Checklist	PwD/CG
Personal and social resources	RUD	CG
Health-related quality of life	EQ-5D-5L	PwD
Quality of life of PwD	DEMQOL-U	PwD
	DEMQOL-U-Proxy	CG
	QOL-AD	PwD, CG
	EQ-5D-5L	CG
Quality of relationship	PAI	PwD
Capability	ICECAP-O	PwD
(un)met needs	CANE	PwD, CG, In
Neuropsychiatric symptoms	NPI-Q	CG
Severity of dementia	CDR	In
Comorbidity	Charlson Index	In
Activities of daily life	IADL	CG
	PSMS	CG
Informal carers		
Social isolation	LSNS-6	CG
Quality of relationship	PAI	CG
Quality of life	CarerQol-7D	CG
Health related quality of life	EQ-5D-5L	CG
Anxiety and depression	HADS	CG
Perseverance time	Single question	CG
Stress	RSS	CG
Capability	ICECAP-O	CG
Control	Locus of control^a^	CG
Sense of coherence	SOC-13	CG
Personal and social resources	RUD	CG

*Pwd* people with dementia, *CG* informal carers, *In* interviewer. Measures which are only assessed at baseline are marked with an ^a^

### Main outcomes

One of the main objectives in Actifcare concerns met and unmet needs. These will be assessed with the Camberwell Assessment of Need for the Elderly (CANE), a tool especially designed to combine opinions regarding needs from people with dementia, informal carers and professionals [10]. The Resource Utilisation in Dementia instrument (RUD) measures service use, and will be completed by the researcher based on information provided by the carer. With the RUD we can obtain information regarding medical resources and informal care resource use [11]. A service use checklist was constructed with input from all participating countries to provide more information on the (non)-use of services and the reasons behind this.

### Measures for people with dementia

Measures for people with dementia include a range of quality of life scales. The Quality of Life- Alzheimer’s Disease scale (QOL-AD) is a reliable and valid scale for people with dementia with a Mini Mental State Examination (MMSE) score above 10 [12]; the same accounts for the DEMQOL. Both the QOL-AD and DEMQOL have a proxy-report version as well [13]. The ICECAP-O is a generic instrument that measures capabilities with preference-based tariffs applicable in health-economic evaluation. This promising tool is expected to more sensitively capture changes resulting from the use of formal care services in the middle stage of dementia than the EuroQol [14]. Health-related quality of life scales will also be administered. The EuroQol-5D has been validated in a number of European countries in and in the dementia population. It consists of five items and a people with mild to moderate dementia and it assesses the subjective perceptions and experiences of people with dementia [13]. In addition cognitive functioning is assessed with the Mini Mental State Examination (MMSE) [15], and the quality of the relationship with the informal carer with the Positive Affect Index (PAI) [16].

### Measures for the informal carers

There are several measures for the informal carer regarding quality of life (EQ-5D-5L, CarerQol, ICECAP-O). The Care related quality of life scale (CarerQol) was developed (along the lines of the EuroQol instrument) to measure the impact of informal care by assessing happiness and describing the most important burden dimensions. This promising instrument will be applied and validated in the participating European countries [17, 18]. Anxiety and depression will be measured with the 14 item Hamilton Anxiety and Depression Scale (HADS) [19]. Perseverance time is measured with a single simple estimate of how long the informal carer can continue in this way if the situation remains unchanged. In addition, caregiving-related stress and social network is assessed with the Relative Stress Scale (RSS) [20] and the Lubben social network scale (LSNS-6) [21]. These measures are important as they give us a broad insight on different aspects of life of the informal carer. Information regarding internal and external locus of control (Locus of Control of Behaviour Scale) and sense of coherence are also assessed (SOC-13) [22, 23].

The carer will also provide information regarding the persons’ with dementia functional abilities. The Instrumental Activities of Daily living (IADL) scale provides us with specific information on daily living skills while the Physical Self-Maintenance Scale (PSMS) gives information about physical abilities [24]. Neuropsychiatric symptoms are assessed with the Neuropsychiatric Inventory (NPI-Q), as these influence caregiver burden [25]. Quality of life will be assessed with several measures similar to those administered with the people with dementia (QOL-AD, EQ-5D-5L, ICECAP-O) along with the DEMQOL-U proxy which is specifically designed for carers to rate quality of life for the people with dementia [13].

### Additional measures

Comorbidities will be assessed with the Charlson Comorbidity Index, to control for service use for causes other than dementia [26]. Severity of dementia will be assessed with the Clinical Dementia Rating (CDR) [27]. People with dementia and carers will also complete a short questionnaire on socio-demographic information (age, gender, ethnicity, education, occupation, living situation).

### Procedures cohort study

At baseline, at 6 months follow-up and at 12-months follow-up all questionnaires will be administered in the hospital or at home to ensure that participants are in a comfortable environment. The visit can be shortened or split in two to reduce the burden for the participants. All researchers involved have been trained in administering the different questionnaires and have clinical experience. In a purposively sampled subgroup of *n* = 10 per country, in-depth semi-structured interviews will be conducted at 12-months follow-up ensuring inclusion of both dyads using formal care services and dyads not using formal care services. The content of the interview is developed from the outcome of a literature review and focus groups. Themes that will be discussed are e.g. attitude towards dementia, cooperation with healthcare professionals, joint decision making of the informal carer and the person with dementia regarding service use. The interviews will be tape-recorded and transcribed verbatim for analysis. Data triangulation through verification by two researchers will be used to ensure the trustworthiness of the data analysis.

### Statistical analyses

Group characteristics per individual country will be calculated with proportions or means. Group comparisons will be performed with Chi square tests for categorical variables and t-tests for continuous variables. Transcultural differences are a specific area of interest. To ensure valid comparison of service use among countries direct standardizations will be carried out (using the entire pooled sample as the external standard population) for the effects of age group, gender, educational level, dementia diagnosis and severity. The relationship between predisposing factors, enabling factors and use of formal care services will be explored with a multi-level analysis. Cross level effect modification (e.g. living in a specific country/country cluster modifies the effect of individual characteristics on service use) will be examined to identify ecological effects. To assess the potential inequity with which services are accessed and delivered, the associations between service use and predisposing and enabling variables will be controlled for needs. Multiple regression analysis will be used to explore the relationship between service use, met and unmet needs (independent variables) and quality of life of the people with dementia and informal carer (dependent variables).

## Discussion

The current study focuses on middle stage dementia, and explores the association between the use of formal care, needs and quality of life in people with dementia and their informal carers in eight European countries. This paper describes the research protocol of the cohort study. The Actifcare projects aims to increase our understanding of why people with dementia and their informal carers use, or fail to use formal care services across Europe, and how the use of formal care is experienced.

The strengths of this cohort study are the overall size, where patient inclusion in different countries enables cross-country comparison. The fact that participants are included across different parts of Europe ensures diversity in the group, which enables us to investigate contextual differences. Measurements are assessed at three different time points; in this way we can follow patients and their carers throughout the trajectory in which formal care is initiated. One of the potential limitations is selection bias; those people who refuse service use are not likely to take part in a study concerning needs and service use, as they refuse all types of interference. It would be interesting to hear the rationale behind decisions from these people with dementia.

With the input of people with dementia, their informal carers and professionals we can develop formal care strategies, and combine these with information on cost-efficiency across Europe. This information will help us develop best-practice strategies to improve effectiveness and efficiency of access to European dementia care systems. We will reach a consensus regarding recommendations across countries, and create country-specific recommendations for the implementation of best practice strategies. Once the project ends and recommendations are developed, we will disseminate these results to a wide audience through different methods. The target audience is health care professionals, national health services, the general public, patient advocacy groups and dementia researchers. A Consortium and Advisory board of expertise has been set up, representing different professional disciplines; several representatives of the project are closely related to national political boards as well as to institutions and political boards of the European Union. This will additionally facilitate widespread dissemination of results. The results will be available after the end of the cohort study in June 2016.

## Abbreviations

Actifcare: ACcess to Timely Formal Care study
CANE: Camberwell assessment of need for the elderly
CarerQol: Care related quality of life scale
CDR: Clinical dementia rating
DEMQOL: Dementia quality of life
HADS: Hamilton anxiety and depression scale
IADL: Instrumental activities of daily living
LSNS-6: Lubben social network scale
MMSE: Mini mental state examination
NPI-Q: Neuropsychiatric inventory
PAI: Positive affect index
PSMS: Physical self-maintenance scale
QOL-AD: Quality of life- Alzheimer’s disease scale
RSS: Relative stress scale
RTCP: Right time place care
RUD: Resource utilisation in dementia instrument
SOC-13: Sense of coherence
