# Erratum to: Access to timely formal dementia care in Europe: protocol of the Actifcare (ACcess to Timely Formal Care) study

**DOI:** 10.1186/s12913-016-1877-5

**Published:** 2016-10-28

**Authors:** Liselot Kerpershoek, Marjolein de Vugt, Claire Wolfs, Hannah Jelley, Martin Orrell, Bob Woods, Astrid Stephan, Anja Bieber, Gabriele Meyer, Knut Engedal, Geir Selbaek, Ron Handels, Anders Wimo, Louise Hopper, Kate Irving, Maria Marques, Manuel Gonçalves-Pereira, Elisa Portolani, Orazio Zanetti, Frans Verhey, the Actifcare Consortium

**Affiliations:** 1 Maastricht University, Maastricht, Netherlands; 2 Bangor University, Bangor, UK; 3 UCL, London, UK; 4 Martin-Luther University Halle-Wittenberg, Halle, Germany; 5 Oslo University Hospital, Oslo, Norway; 6 Karolinska Institutet, Solna, Sweden; 7 Dublin City University, Dublin, Ireland; 8 CEDOC, Nova Medical School | Faculdade de Ciências Médicas, Universidade Nova de Lisboa, Lisbon, Portugal; 9 Alzheimer’s Research Unit-Memory Clinic, IRCCS “Centro S.Giovanni di Dio, Brescia, Italy

## Erratum

After publication of the original article [1] it was brought to our attention that author Martin Orrell was incorrectly included as Martin Orrel. The correct spelling of the name is included in the author list of this erratum and updated in the original article.
